# Automated thresholding algorithms outperform manual thresholding in macular optical coherence tomography angiography image analysis

**DOI:** 10.1371/journal.pone.0230260

**Published:** 2020-03-20

**Authors:** Jan Henrik Terheyden, Maximilian W. M. Wintergerst, Peyman Falahat, Moritz Berger, Frank G. Holz, Robert P. Finger

**Affiliations:** 1 Department of Ophthalmology, University of Bonn, Bonn, Germany; 2 Department of Medical Biometry, Informatics and Epidemiology, University of Bonn, Bonn, Germany; Nicolaus Copernicus University, POLAND

## Abstract

**Introduction:**

For quantification of Optical Coherence Tomography Angiography (OCTA) images, Vessel Density (VD) and Vessel Skeleton Density (VSD) are well established parameters and different algorithms are in use for their calculation. However, comparability, reliability and ability to discriminate healthy and impaired macular perfusion of different algorithms are unclear, yet, of potential high clinical relevance. Hence, we assessed comparability and test-retest reliability of the most common approaches.

**Materials and methods:**

Two consecutive 3×3mm OCTA en face images of the superficial and deep retinal layer were acquired with swept-source OCTA. VD and VSD were calculated with manual thresholding and six automated thresholding algorithms (Huang, Li, Otsu, Moments, Mean, Percentile) using ImageJ and compared in terms of intra-class correlation coefficients, measurement differences and repeatability coefficients. Receiver operating characteristic analyses (healthy vs. macular pathology) were performed and Area Under the Curve (AUC) values were calculated.

**Results:**

Twenty-six eyes (8 female, mean age: 47 years) of 15 patients were included (thereof 15 eyes with macular pathology). Binarization thresholds, VD and VSD differed significantly between the algorithms and compared to manual thresholding (p < 0.0001). Inter-measurement differences did not differ significantly between patients with healthy versus pathologic maculae (p ≥ 0.685). Reproducibility was higher for the automated algorithms compared to manual thresholding on all measures of reproducibility assessed. AUC was significantly higher for the Mean algorithm compared to the manual approach with respect to the superficial retinal layer.

**Conclusions:**

Automated thresholding algorithms yield a higher reproducibility of OCTA parameters and allow for a more sensitive diagnosis of macular pathology. However, different algorithms are not interchangeable nor results readily comparable. Especially the Mean algorithm should be investigated in further detail. Automated thresholding algorithms are preferable but more standardization is needed for clinical use.

## Introduction

Optical coherence tomography angiography (OCTA) provides depth resolved high resolution images of the retinal and choroidal blood flow. [1,2] A number of different approaches are available to quantify OCTA image data, but to date both their reproducibility as well as their comparability are unclear.

Image processing is a crucial step when generating comparable and reliable quantitative data from retinal images. For the calculation of global vessel density (VD) from OCTA images, definition of a threshold for image binarization is essential. The three most common solutions are manual binarization methods, automated binarization methods using open source software and automated binarization using commercial software. [3–9] Manual and semi-automated methods often gain a threshold for binarization based on the signal within the vessel-free foveal avascular zone (FAZ). Automated algorithms use e.g. the histogram of a complete OCTA image or local clusters to obtain a threshold. Advantages of manual/semiautomated and automated binarization methods, as compared to commercial software, include high transparency for research purposes. Besides these two options, commercial software is available from various device manufacturers as well as other sources, mostly using proprietary image processing algorithms not publicly available. Additionally, fixed threshold and machine learning approaches are available. [10,11]

A recent study by Rabiolo and colleagues [12] found significant differences in VD calculations between manual and automated approaches but used arbitrary cut-offs for their manual binarization and did not assess test-retest reliability of two consecutive examinations. Thus, in this study we assessed both repeatability and comparability of manual binarization based on the FAZ and six automated algorithms for OCTA image binarization in patients with and without macular disease.

## Materials and methods

### Subject recruitment

Participants both healthy and with any macular pathology impairing the vasculature were consecutively recruited at the Department of Ophthalmology, University of Bonn, Germany, between April and August 2018. Ethical approval was obtained from the ethics committee of the University of Bonn (approval ID 089/08) and informed consent was obtained from all study participants prior to study initiation after explanation of the nature and possible consequences of the study. The study was conducted in adherence to the tenets of the Declaration of Helsinki. Exclusion criteria were artefacts diminishing image quality, OCTA signal strength index < 7, inability to fixate and clinically relevant media opacities.

### Image acquisition

Two OCTA images per eye were consecutively obtained using a swept-source OCTA device (Zeiss PLEX Elite 9000, Carl Zeiss Meditec, Dublin, California, USA) with 100,000 A-scans per second (central wavelength: 1040-1060 nm). The scan size of all scans was 3×3mm with focus on the macula. B-scans were segmented automatically using the automated algorithm of the device and manually reviewed for segmentation errors. The proprietary general sliding slab method of the device was used to remove decorrelation tail artefacts within the OCTA volume (Bagherinia H, et al., IOVS 2017;58:ARVO E-Abstract 643). According to the current OCTA nomenclature, en face images of the superficial retinal layer (encompassing nerve fibre layer, ganglion cell layer, inner plexiform layer) and deep retinal layer (inner nuclear layer, outer plexiform layer, Henle fibre layers) were exported [13].

### Image analysis

Fiji [14], an open-source image processing software based on ImageJ [15] (version 1.51w) was used for image analysis. Per eye, two 8-bit grey scale en face bitmap images of the superficial and deep retinal layers were binarized by manual thresholding and six previously published automated algorithms [16–21] implemented in Fiji, named as followed, based on the abbreviations used in the software[15]: Huang, Li, Otsu, Moments, Mean and Percentile. As previously described, the manual approach was based on delineating the FAZ by selecting its outer borders with a free-hand tool on the superficial retinal layer and using the maximum grey value in this area as the threshold for binarization. [3,8,22–28] A second measurement was performed after at least 1 week by the same examiner, in case of a grey value threshold difference between the 2 measurements ≥ 5, a third measurement was performed immediately and the median of these 3 measurements used as the threshold for the manual method. It has been shown that this methodology has high interrater reliability. [3] For the deep retinal layer, the FAZ selection of the superficial retinal layer was applied to the respective image area of the deep layer for binarization threshold determination. This approach combines best practice manual thresholding approaches published in the literature. [3,22] The threshold determination of the six automated algorithms has been previously published elsewhere. [16–21] VD was calculated based on the binarized images according to the formula $VD=\frac{{n(white\;pixels\;in\;binarized\;image)}^{2}}{{n(all\;pixels\;in\;binarized\;image)}^{2}}$. [22] For calculation of Vessel Skeleton Density (VSD) images were skeletonized by ImageJ and VSD was calculated according to the formula $VSD=\frac{n(white\;pixels\;in\;skeletonized\;image)}{{n(all\;pixels\;in\;skeletonized\;image)}^{2}}$. [22]

### Statistical analyses

Statistical analyses were performed with SPSS Statistics for Windows, version 25 (IBM Corporation, Armonk, New York). Mean values of all VD and VSD measurements per layer and per eye were calculated and tested for associations with signal strength index of the OCTA image, intraocular pressure and patient age. The relative differences between test and retest VD and VSD were calculated. Linear regression analysis was performed to adjust for age, including relative differences between test and retest VD and VSD values as dependent variables and age as well as seven binary variables for the respective algorithm used as independent variables. Intra-class correlation coefficients (ICCs) between the two OCTA images of each eye were determined. Additionally, the Repeatability Coefficient (RC) was calculated according to the formula $RC=1.96\times \sqrt{\frac{{\sum \left(measurement\;2-measurement\;1\right)}^{2}}{n}}\;$. [29–31] The Mann-Whitney-U test, the Friedman test and the Kruskal Wallis test were used as indicated. To measure discriminatory ability (healthy versus macular pathology), differences between the different approaches were assessed using Receiver Operating Characteristic (ROC) curves and area under the curve (AUC) values. For age adjustment, we performed a binary logistic regression analysis to discrminate between healthy eyes and eyes with a macula based on VD or VSD and age (per algorithm and per retinal layer). The resulting probabilities were then used for ROC analysis. A p-value of <0.05 was considered statistically significant. Correction for multiple testing was done using the Holm-Bonferroni method [32]. Corrected p-values are reported as p_c_.

## Results

Twenty-six eyes (8 female, 18 male; mean age: 47 years) of 15 patients were included, resulting in 104 images (two consecutive images of two layers per eye) of the superficial and deep retinal layer. These included 11 healthy eyes and 15 eyes with an impaired macular vasculature (due to diabetic changes, previous central venous occlusions or other maculopathies). Some eyes had to be excluded due to insufficient image quality. Sphere ranged between ± 2.0 dpt in all eyes. Intraocular pressure was within a normal range in all patients (mean ± standard deviation: 17 mmHg ± 4 mmHg). In all included images, the OCTA en face image quality was high with a minimum signal strength index of 9/10.

**Fig 1:**
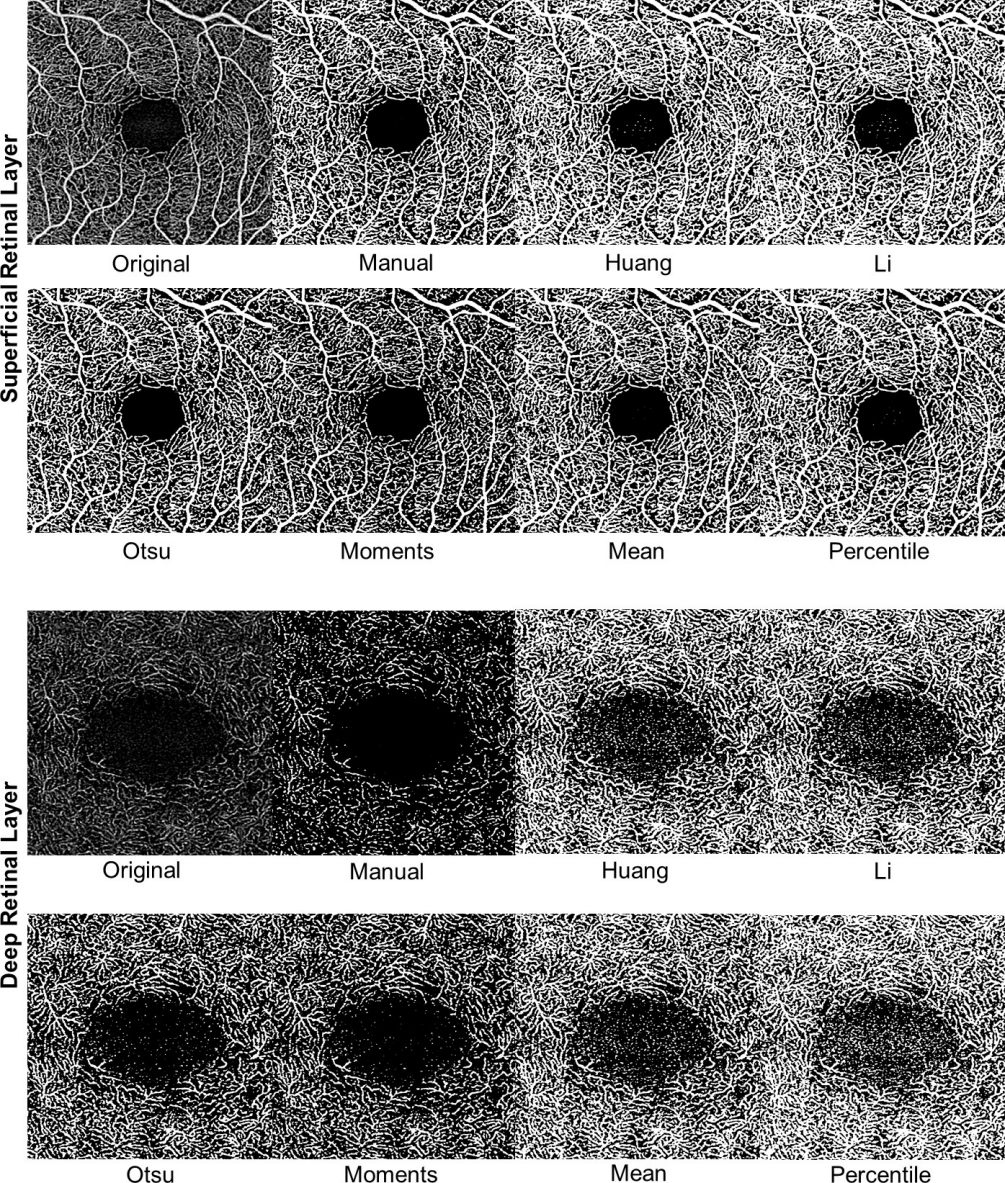
OCTA en face images of the superficial and deep retinal layers of one healthy eye. The image order from left to right is: Original image, binarized images using the manual thresholding and the Huang, Li, Otsu, Moments, Mean, Percentile algorithms.

The average threshold values differed significantly between different binarization approaches (p < 0.0001, Table 1). Overall, the Moments algorithm produced the highest and the Percentile and Li algorithms the lowest mean thresholds. Overall binarization thresholds did not differ significantly between subjects with healthy maculae and subjects with macular vessel pathology (p = 0.447). Exemplary unprocessed and binarized images are displayed in **Fig 1**.

**Table 1:** Mean ± standard deviation of binarization thresholds, vessel densities and skeleton densities obtained using the different algorithms for images of the superficial and deep retinal layers.

Parameter	Layer	Binarization approach						
		Manual	Huang	Li	Otsu	Moments	Mean	Percentile
Binarization threshold	Superf.	63 ± 12	49 ± 4	49 ± 4	66 ± 8	74 ± 10	58 ± 7	52 ± 9
	Deep	56 ± 13	30 ± 5	30 ± 4	41 ± 7	45 ± 9	32 ± 4	26 ± 5
Vessel Density	Superf.	0.196 ± 0.089	0.276 ± 0.058	0.149 ± 0.024	0.163 ± 0.035	0.120 ± 0.025	0.206 ± 0.025	0.250 ± 0.003
	Deep	0.052 ± 0.037	0.190 ± 0.034	0.197 ± 0.038	0.102 ± 0.034	0.079 ± 0.027	0.174 ± 0.024	0.249 ± 0.005
Skeleton Density	Superf.	6.1×10^-8^ ± 1.4×10^-8^	7.0×10^-8^ ± 1.0×10^-8^	7.1×10^-8^ ± 1.0×10^-8^	5.9×10^-8^ ± 0.9×10^-8^	5.3×10^-8^ ± 0.8×10^-8^	6.4×10^-8^ ± 0.7×10^-8^	6.9×10^-8^ ± 0.5×10^-8^
	Deep	3.5×10^-8^ ± 1.4×10^-8^	6.6×10^-8^ ± 0.7×10^-8^	6.7×10^-8^ ± 0.7×10^-8^	4.9×10^-8^ ± 0.9×10^-8^	4.4×10^-8^ ± 0.9×10^-8^	6.4×10^-8^ ± 0.5×10^-8^	7.5×10^-8^ ± 0.3×10^-8^

Superf. = superficial

VD and VSD differed significantly between different binarization algorithms (p < 0.0001, **Table 1**). Both values were significantly lower in individuals with macular pathology compared to those without (p < 0.0001). For instance, mean VD of the superficial layer was 0.22 ± 0.01 for healthy eyes and 0.19 ± 0.03 for diseased eyes while VSD of the superficial layer was 6.9×10^-8^ ± 0.3×10^-8^ for healthy eyes and 6.1×10^-8^ ± 0.8×10^-8^ for diseased eyes according to the Mean algorithm. VD and VSD did not differ significantly between different image signal strength indices (p = 0.157 and p = 0.079, respectively) or intraocular pressures (p = 0.271 and p = 0.462, respectively). Age was negatively correlated with VD (r = -0.405, p = 0.040) and VSD (r = -0.406, p = 0.039).

**Fig 2:**
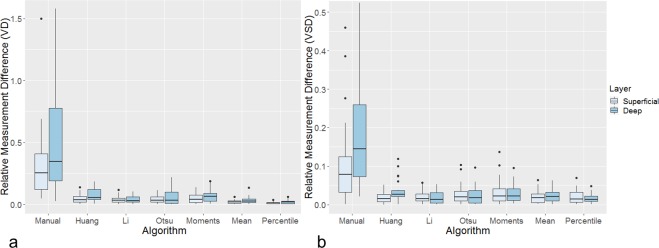
Inter-algorithm repeatability: Relative Vessel Density (a) and Vessel Skeleton Density (b) measurement differences obtained using a manual approach and six automated algorithms to binarize images of the superficial and deep retinal layers. Some outliers are not shown due to the scaling of the diagram. Values over 1.5 interquartile range below the first quartile or above the third quartile were defined as outliers; the horizontal lines indicate the 1^st^ quartile, median and 3^rd^ quartile. VD = Vessel Density, VSD = Vessel Skeleton Density.

Relative test-retest measurement differences of VD and VSD obtained from two consecutive OCTA examinations varied significantly between the algorithms when comparing all approaches using the Friedman test both in the superficial and deep retinal layers (p < 0.0001). The manual thresholding approach had a significantly lower VD and VSD repeatability in the superficial and deep retinal layer compared to most automated algorithms in pair-wise comparisons between the different binarization approaches (p_c_ < 0.0084 in paired algorithm comparisons, **Fig 2**). The relative inter-measurement differences of the Huang algorithm (VD, deep retinal layer: p_c_ = 0.126; VSD, superficial and deep retinal layer: p_c_ ≥ 0.064) and the Li algorithm (VSD, superficial retinal layer: p_c_ = 0.183) did not differ significantly from the repeatability values of the manual method. The relative inter-measurement differences, however, did not differ significantly when comparing automated algorithms with one another (p_c_ > 0.05). To adjust for age, linear regression analysis was performed. For both VD and VSD, the relative test-retest differences significantly depended on use of the manual approach compared to not using it (β = 0.661, p < 0.0001 and β = 0.163, p < 0.0001, respectively). The VD and VSD relative test-retest differences did not significantly depend on age or use of any of the automated algorithms. Inter-examination VD and VSD differences between algorithms were not significantly different between healthy eyes and eyes with macular vessel pathologies (VD, p = 0.685; VSD, p = 0.770), indicating that the results of both groups can be interpreted in total.

A post-hoc power analysis for the Friedman test using a 10000-fold simulation with data generated from a normal distribution according to our sample characteristics revealed that a sample size of 6 eyes is sufficient to detect significant differences at 5% level with 80% power between VD relative differences of different algorithms in the superficial and in the deep retinal layers. Therefore, the sample size available in our study was considered appropriate from a statistical standpoint.

**Fig 3:**
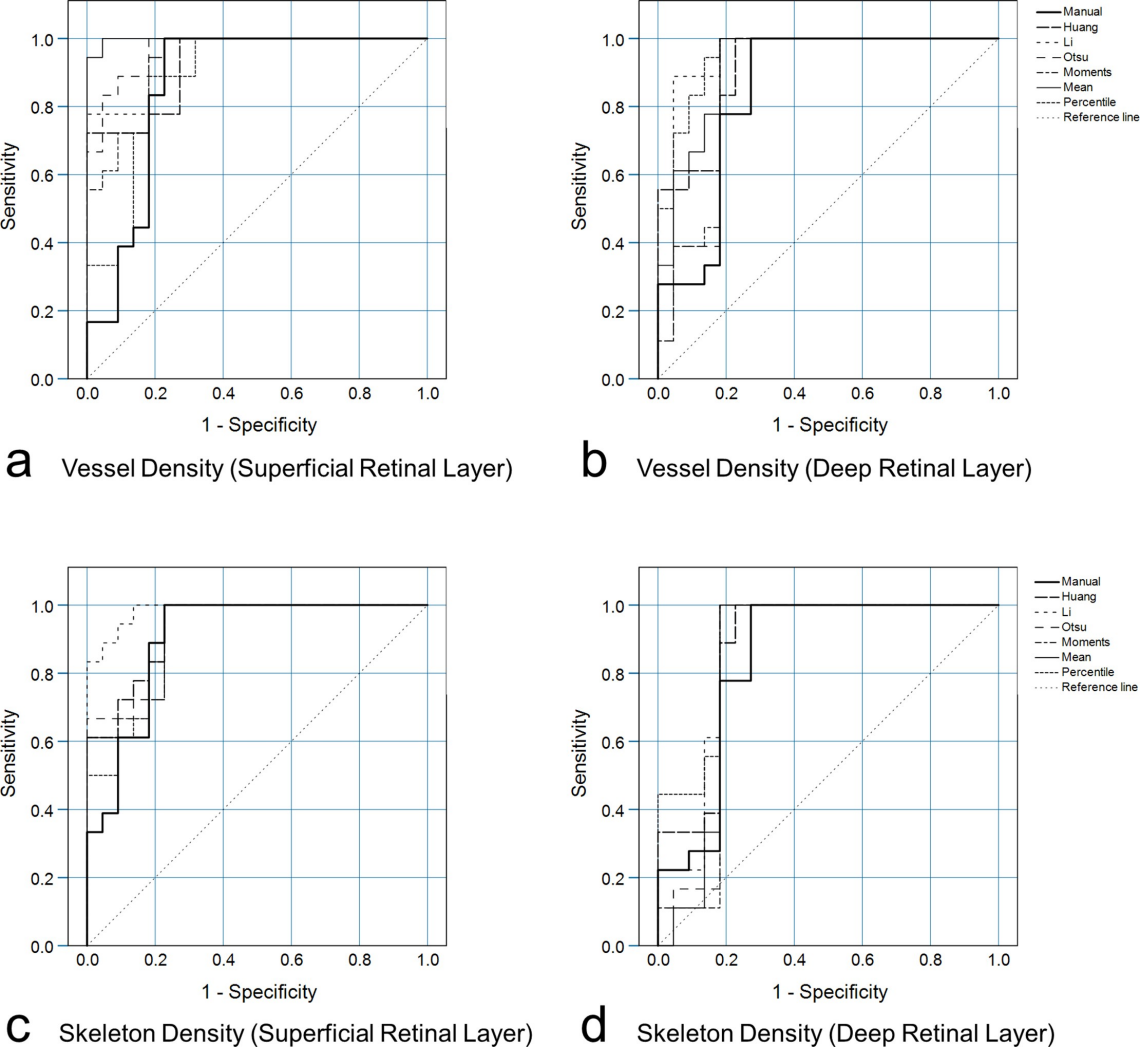
ROC curves (healthy versus macular pathology) of the binary logistic regression formulae based on the respective Vessel Density and Vessel Skeleton Density as well as age obtained by the manual approach and the automated algorithms in the superficial (a, c) and deep retinal layer (b, d).

Except for the percentile algorithm, ICCs of VD and VSD measurements between the two consecutive examinations per eye were noticeably higher for all the automated algorithms when compared to the manual approach (**Table 2)**. Repeatability Coefficients of VD and VSD of the superficial and deep retinal layers were noticeably higher (i.e. poorer repeatability) for the manual approach compared to the six automated algorithms investigated (**Table 2**). ROC analysis (healthy versus macular pathology) based on binary logistic regression models to adjust for age revealed AUC values between 0.838 and 0.997. The manual method was less sensitive to pathologic change than most automated algorithms (**Fig 3**) and the four AUC values of the manual approach were lower than almost all (23/24, 96%) values of automated algorithms. The AUC value for the manual method of VD acquisition of the superficial retinal layer was significantly lower (indicating lower sensitivity and specificity values) than the AUC value for the Mean algorithm of VD acquisition of the same layer (**Table 3**).

**Table 2:** Additional repeatability parameters.

Parameter		Layer	Repeatability value per Algorithm						
			Manual	Huang	Li	Otsu	Moments	Mean	Percentile
ICC [95% CI]	Vessel Density	Superf.	0.625 [0.163–0.832]	0.907 [0.793–0.958]	0.829 [0.618–0.923]	0.974 [0.942–0.988]	0.949 [0.885–0.977]	0.949 [0.886–0.977]	-0.070[-1.386–0.520]
		Deep	0.735 [0.410–0.881]	0.812 [0.581–0.916]	0.929 [0.842–0.968]	0.942 [0.870–0.974]	0.957 [0.904–0.981]	0.966 [0.924–0.985]	0.096 [-1.017–0.595]
	Skeleton Density	Superf.	0.802 [0.559–0.911]	0.981 [0.957–0.991]	0.979 [0.953–0.991]	0.986 [0.969–0.994]	0.982 [0.961–0.992]	0.984 [0.965–0.993]	0.884 [0.742–0.948]
		Deep	0.643 [0.203–0.840]	0.854 [0.675–0.935]	0.977 [0.948–0.990]	0.942 [0.871–0.974]	0.947 [0.881–0.976]	0.945 [0.878–0.976]	0.305 [-0.550–0.688]
RC	Vessel Density	Superf.	0.191	0.026	0.024	0.016	0.016	0.009	0.007
		Deep	0.062	0.033	0.018	0.016	0.012	0.014	0.012
	Skeleton Density	Superf.	2.2×10^-8^	3.1×10^-9^	3.4×10^-9^	3.9×10^-9^	4.3×10^-9^	3.0×10^-9^	3.4×10^-9^
		Deep	1.9×10^-8^	5.4×10^-9^	3.1×10^-9^	3.2×10^-9^	3.1×10^-9^	3.4×10^-9^	3.0×10^-9^

CI = confidence interval; ICC = Intra-class correlation coefficient; p_c_ = corrected p-value; RC = Repeatability Coefficient; Superf. = superficial

**Table 3:** Results of ROC analysis of binary logistic regression formulae based on the respective Vessel Density and Vessel Skeleton Density as well as age in the superficial and deep retinal layers per algorithm.

Variable	Layer	Area Under the Curve per Algorithm [95% Confidence Interval]						
		Manual	Huang	Li	Otsu	Moments	Mean	Percentile
Vessel Density	Superf.	0.864 [0.741;0.986]	0.929 [0.855;1.0]	0.957 [0.903;1.0]	0.967 [0.922;1.0]	0.934 [0.863; 1.0]	0.997 [0.989;1.0]	0.884 [0.778;0.989]
	Deep	0.851 [0.727;0.975]	0.917 [0.833;1.0]	0.965 [0.914;1.0]	0.876 [0.758;0.994]	0.879 [0.763;0.995]	0.927 [0.846;1.0]	0.955 [0.896;1.0]
Skeleton Density	Superf.	0.902 [0.806;0.998]	0.934 [0.864;1.0]	0.985 [0.958;1.0]	0.929 [0.855;1.0]	0.922 [0.842;1.0]	0.922 [0.842;1.0]	0.909 [0.821;0.997]
	Deep	0.843 [0.714;0.973]	0.876 [0.762;0.990]	0.876 [0.757;0.996]	0.864 [0.706;0.986]	0.838 [0.692;0.985]	0.843 [0.701;0.986]	0.884 [0.782;0.986]

Superf. = superficial

## Discussion

To our knowledge this is the first quantitative study on test-retest reliability of manual versus automated thresholding approaches for OCTA images. Automated algorithms outperformed manual thresholding, lead to more reproducible results and, therefore, allow for a more sensitive discrimination of healthy maculae from maculae with pathology. Thus, automated binarization algorithms should be preferred over manual approaches for OCTA image analysis. However, the different algorithms are not interchangeable and results of the algorithms differed significantly. Therefore, a better – ideally international – standardization of algorithms is needed to increase comparability of studies.

Our study supports existing data showing low inter-method agreement for image binarization. [12,33] The thresholds between the algorithms varied significantly as did the VD and VSD values, highlighting a lack of comparability. Rabiolo and colleagues also compared different methods to quantify perfusion in the macular region, however, they only assessed VD, used lower resolution 6×6mm en face OCTA images and did not investigate test-retest reliability of the different approaches. [12] Mehta and colleagues recently applied five automated binarization algorithms to OCTA images and found significant differences between the detected VD values. However, they investigated neither the repeatability of VD values based on the algorithms nor their ability to detect pathology. [33] Shoji and colleagues compared different automated thresholding algorithms but they did not assess any manual methods. [34] In this study, we used high-detail 3×3mm en face OCTA images, provide data on VD as well as VSD and evaluated test-retest reliability of both manual and automated algorithms on consecutive images.

Reproducibility was excellent for five of the automated algorithms according to the scale proposed by Chan. [35] This is in keeping with the current literature where automated algorithms tend to outperform manual image analysis in terms of reproducibility. [36–38] The inter-measurement differences were significantly higher after image binarization using the manual algorithm compared to all automated algorithms. This effect was independent of age. Due to this, automated binarization algorithms should be preferred over manual approaches in OCTA image binarization.

Intra-class correlation coefficients between the two consecutive OCTA measurements were significantly higher when using the Otsu, Moments or Mean algorithms compared to the manual approach in at least three out of four categories investigated (VD and VSD of the superficial and deep retinal layers, respectively). For this reason, these three algorithms should be investigated in further detail. The automated Percentile algorithm proved not to be appropriate for analysis of VD or VSD due to inconsistent results. It measures the grey intensity closest to a percentile which limits the use of this algorithm when applied to OCTA (Table 4). We therefore do not recommend the Percentile Algorithm for future investigation on this topic.

**Table 4:** Principles of the binarization methods used in this study.

Binarization method	Short description [3–9]
Manual	Determines maximum gray value within the marked foveal avascular zone
Huang	Minimizes fuzziness of pixel-wise fuzzy membership functions
Li	Minimizes cross entropy between unedited and binarized images
Otsu	Minimizes variance between foreground and background structures in the image histogram
Moments	Preserves gray-level moments of the input image
Mean	Calculates mean of grey levels in the original image
Percentile	Measures the grey intensity closest to a percentile

Ability to discriminate between healthy and pathological maculae was good for almost all automated algorithms except the Percentile algorithm. Rabiolo et al found no significant differences between the algorithms tested in their study. [12] Interestingly, the manual approach used by Rabiolo and colleagues discriminated healthy individuals from those with macular disease noticeably better than our manual approach. However, information on reliability of their manual approach is missing. Our method for manual thresholding was standardized against the FAZ whereas Rabiolo et al used arbitrary binarization thresholds. Thus it is doubtful whether their results can be reproduced with other images and manual thresholding cannot be recommended. Shoji et al compared different automated global and local thresholding methods across two OCTA devices [34]. They also included the Otsu and the Mean algorithms in their analysis but reported significant lower intra-class correlations compared to our data (based on the 95% confidence intervals). Our work additionally included age-adjusted statistical comparisons of different algorithms, showing that the Mean algorithm can be used to detect pathology significantly better that the manual approach. Such data have not been available in the previous literature. According to our results, specifically the Mean algorithm might be a good option to use for binarization of en face OCTA images of the superficial retinal layer with the PLEX Elite device. It tends to enhance the retinal vasculature compared to other algorithms as reflected in relatively high mean VD and VSD values. For this reason, it might be more prone to image artefacts than algorithms that overall set higher binarization thresholds like the Otsu or Moments algorithms.

The strengths of our study include a comprehensive evaluation of reliability and comparability of seven different approaches for OCTA image binarization, all previously published, including four measures of repeatability. We also assessed ability to discriminate healthy from diseased eyes for all approaches. For image acquisition, well established protocols were used, allowing for easy replication of our study. In the literature, more comprehensive preprocessing steps for image processing have been proposed, including the use of filters such as the Frangi filter to enhance image contrasts. We have omitted such steps in our analysis on purpose, since these methods have several disadvantages including generation of image artifacts resembling vessel structures and different results for vessels that are not equally distributed in size [39,40]. Limitations include the relatively small number of subjects (therefore limited generalizability of the comparisons between different automated algorithms), having only one repeat measurement per eye and limited comparability of vessel density and skeleton density values because of different calculation approaches in the literature. We did not assess comparability and reproducibility across different OCTA devices and evaluated OCTA scans of the macula only. We did not compare the algorithms to commercial tools for quantification of vessel parameters because for scientific purposes, understanding as many of the image processing steps as possible is warranted. Future research should also focus on binarization of OCTA images of the optic disc with different algorithms.

In conclusion, because of higher repeatability and improved discrimination, automated binarization algorithms should be preferred over manual approaches. Better standardization of algorithms is needed to improve comparability of studies.
